# Anthropometric Variables Accurately Predict Dual Energy X-Ray Absorptiometric-Derived Body Composition and Can Be Used to Screen for Diabetes

**DOI:** 10.1371/journal.pone.0024017

**Published:** 2011-09-06

**Authors:** Reza Yavari, Erin McEntee, Michael McEntee, Michael Brines

**Affiliations:** 1 Beyond Care, LLC, Guilford, Connecticut, United States of America; 2 Department of Internal Medicine, Yale School of Medicine, Yale University, New Haven, Connecticut, United States of America; 3 Physician Assistant Program, School of Medicine and Health Sciences, The George Washington University, Washington, D.C., United States of America; 4 Impact Health Biometric Testing, Inc., King of Prussia, Pennsylvania, United States of America; 5 Research and Development, The Kenneth S. Warren Institute, Ossining, New York, United States of America; Universita Magna-Graecia di Catanzaro, Italy

## Abstract

The current world-wide epidemic of obesity has stimulated interest in developing simple screening methods to identify individuals with undiagnosed diabetes mellitus type 2 (DM2) or metabolic syndrome (MS). Prior work utilizing body composition obtained by sophisticated technology has shown that the ratio of abdominal fat to total fat is a good predictor for DM2 or MS. The goals of this study were to determine how well simple anthropometric variables predict the fat mass distribution as determined by dual energy x-ray absorptometry (DXA), and whether these are useful to screen for DM2 or MS within a population. To accomplish this, the body composition of 341 females spanning a wide range of body mass indices and with a 23% prevalence of DM2 and MS was determined using DXA. Stepwise linear regression models incorporating age, weight, height, waistline, and hipline predicted DXA body composition (i.e., fat mass, trunk fat, fat free mass, and total mass) with good accuracy. Using body composition as independent variables, nominal logistic regression was then performed to estimate the probability of DM2. The results show good discrimination with the receiver operating characteristic (ROC) having an area under the curve (AUC) of 0.78. The anthropometrically-derived body composition equations derived from the full DXA study group were then applied to a group of 1153 female patients selected from a general endocrinology practice. Similar to the smaller study group, the ROC from logistical regression using body composition had an AUC of 0.81 for the detection of DM2. These results are superior to screening based on questionnaires and compare favorably with published data derived from invasive testing, e.g., hemoglobin A1c. This anthropometric approach offers promise for the development of simple, inexpensive, non-invasive screening to identify individuals with metabolic dysfunction within large populations.

## Introduction

Currently, a pandemic of obesity exists that is associated with a number of serious and debilitating diseases, including diabetes mellitus type 2 (DM2) and metabolic syndrome (MS), a condition which is characterized by central obesity, glucose intolerance, hypertension, and hyperlipidemia [1]. The economic, cultural, and personal costs of these conditions are extremely high and are projected to grow rapidly in the future. For example, currently about 25% of North Americans have MS [2] and its presence doubles the risk of adverse cardiovascular outcomes [3]. Further, results of a number of prospective studies show that identification of individuals at high risk for metabolic disease allows for direct life style or medical interventions that delay or prevent the progression of DM2 [4], [5] and MS [6], [7]. However, to date reliable identification of individuals with undiagnosed metabolic dysfunction within a population has required invasive blood sample collection and laboratory testing, e.g., fasting serum glucose concentrations, oral glucose tolerance testing (OGTT), or hemoglobin A1c determination. Clearly, because the prevalence of DM2 and MS within large populations in the developed and developing world countries is so high, there is a need for simple, effective screening methods to identify those individuals who likely have disease. Those at high risk could then be offered follow-up diagnostic testing to determine definitively the degree of metabolic dysfunction and offered appropriate treatments.

There has been an intense interest focused on the potential pathophysiologic pathway(s) by which obesity causes disease. Notably, the mere presence of obesity is not a sensitive measure of risk for metabolic dysfunction, as an appreciable number of obese individuals within a population do not currently have disease, and furthermore, will not progress into a prediabetic state. Abundant research effort has focused on identifying what specific aspects of obesity are important predictors of the eventual development of cardiometabolic risk. One promising lead has focused on the pattern of regional fat distribution. On the basis of abundant investigational work, waist circumference [8] and intra-abdominal (i.e., trunk or visceral) fat have been identified to be important predictors of disease [9], and the latter is also hypothesized to be a direct cause of the development of a glucose intolerance and frank diabetes. Notably, individuals with increased central fat depots have increased circulating levels of proinflammatory cytokines and other inflammatory molecules (e.g., C-reactive protein) that are associated with insulin resistance, cardiovascular disease, lipid abnormalities, and hypertension.

From the perspective of public health screening for individuals at risk for metabolic disease, estimation of body fat distribution using the available methods to accurately determine body composition, i.e., computerized tomography, magnetic resonance imaging, or dual energy x-ray absorptometry (DXA), are generally impractical for field use. It is therefore desirable to have available simple methods that can accurately estimate body composition. With respect to this need, it is obvious that the body shape and size of individual humans are more similar than different. It is also logical to assume that as weight increases with the deposition of fat, muscle mass must increase proportionally to effectively support incremental increases in weight. It is likely, therefore, that fat mass (FM) and fat free mass (FFM) depend upon weight in a predictable manner. Several investigators employing isotopic dilution methodology have studied subjects of a wide range of body masses to determine how body composition varies with weight. The results show that a predictable mathematical relationship exists such that FFM is proportional to logarithm of FM [10], [11]. However, these studies did not take into account regional differences of fat deposition, which are likely important as trunk fat is specifically associated with lean muscle mass, due in part to the effects of gravity [12].

Abundant previous work that has also shown that body composition as determined by isotope dilution or hydrological methods to determine body density, i.e., FM and FFM, can be reasonably estimated by anthropometrics over a wide range of body mass in both restricted [13] and generalized populations [14]. In recent years, the development of DXA has allowed for rapid determinations of body composition with a high precision comparable to the results of hydrostatic weighing, and DXA methodology also accurately determines the proportion of abdominal fat to total fat [15], [16]. We therefore postulated that simple anthropometric variables can predict the regional distribution of fat and lean tissue masses as determined by DXA. To explore this possibility, we modeled DXA-derived data obtained by a single investigator using anthropometric variables obtained from a group of 341 females spanning a broad range of body shapes and weights. This group was split into two unequal smaller groups and multiple linear regression performed on the larger subset to develop equations that related anthropometric variables to DXA-obtained body composition. The subsequent equations were then tested in a smaller group for cross-validation.

Additionally, since DXA body composition obtained from a longitudinal study of a typical population of North American females can be used to assess risk that an individual will eventually develop DM2 [17], we hypothesized that anthropometrically-determined body composition in the same study group can be used to screen a population to detect individuals at high risk for having DM2 or MS. Finally, we used the predictive equations derived from the DXA study group to determine whether the same equations would predict the presence of DM2 and MS in a different group of females selected from a general endocrinology practice (n = 1153).

## Results

As summarized in Table 1, the study groups were well-matched, spanning a wide range of fat masses from very lean to morbidly obese. Although the prevalence of DM2 was significantly lower in the Index Group (IG; 5%), it was equivalent in the cross validation (CV) and endocrine practice (EP) groups at 13% and 10% respectively. The prevalence of MS did not differ across the groups, ranging from 19% in the EP group to 26% in the CV group. Linear correlations between the anthropometric variables and the DXA-derived body compartments or weight of all women undergoing DXA scanning (IG+CV = 341) are summarized in Tables 2 and 3. Not surprisingly, weight was significantly and strongly correlated with hipline and waistline, moderately with height, but not with age (Table 2). Partial correlations were calculated to estimate the linear association between pairs of variables, i.e., by holding all other variables constant. Partial correlation analysis revealed that waistline had highest correlation with weight. Interestingly, correlations between anthropometric variables and DXA-derived body compartments showed that waistline was not correlated to the total fat mass, but rather to TF and to FFM (Table 2). Although hipline was strongly correlated to FM, TF, and FFM, partial correlation showed that the relationship actually depended on other variables, as the correlation vanished for TF and FFM. As expected, age was only correlated with FFM, which was in an inverse manner.

**Table 1:** Characteristics of the study populations.

group (n)	IG (246)	CV (95)	EP (1153)
Age [Mean ± SD] (Range)	47.7±11.7 (15–73)	46.1±12.1 (16–67)	46.2±11.6 (18–80)
Weight (kg)	85.2±19.2 (42.2–146.2)	86.0±20.7 (45.3–131.6)	87.8±22.2 (43.1–190.5)
Height (m)	1.61±0.08 (1.27–1.83)	1.62±0.07 (1.24–1.8)	1.63±0.08 (1.35–1.92)
DXA FFM (kg)	50.2±8.0 (28.4–71.7)	50.6±8.7 (34.5–76.9)	NA
DXA FM (kg)	34.2±11.8 (8.8–74.4)	35.2±13.3 (10.7–63.2)	NA
BMI	28.8±6.4 (15.6–59.9)	29.2±7.1 (16.8–60.2)	33.1±7.9 (18.2–64.2)
DM2 n (%)	12 (5*)	12 (13)	113 (10)
MS n (%)	52 (21)	27 (27)	219 (19)

*p<0.02 compared to other groups.

**Table 2:** Pearson partial correlation coefficients of anthropometric variables and weight for the full DXA data set (IG+CV = 341 patients).

	weight
**age**	NS
**height**	0.29 (0.37)
**waistline**	0.86 (0.74)
**hipline**	0.76 (0.51)

Value in parentheses is the partial correlation coefficient, i.e., the correlation between two variables with all other variables held constant; NS = non-significant correlation.

**Table 3:** Pearson partial correlation coefficients between anthropometric variables and DXA-derived body compartments for the full DXA data set (n = 341).

	fat mass	trunk fat mass	fat free mass
**age**	NS	NS (0.14)	NS (−0.26)
**height**	0.17 (NS)	0.13 (−0.16)	0.42 (0.48)
**waistline**	0.84 (NS)	0.87 (0.35)	0.74 (0.29)
**hipline**	0.77 (0.33)	0.74 (NS)	0.62 (NS)

Values in parentheses are the partial correlation coefficients. Correlations ≤0.10 or with p>0.05 are considered non-significant (NS).

**Figure 1:**
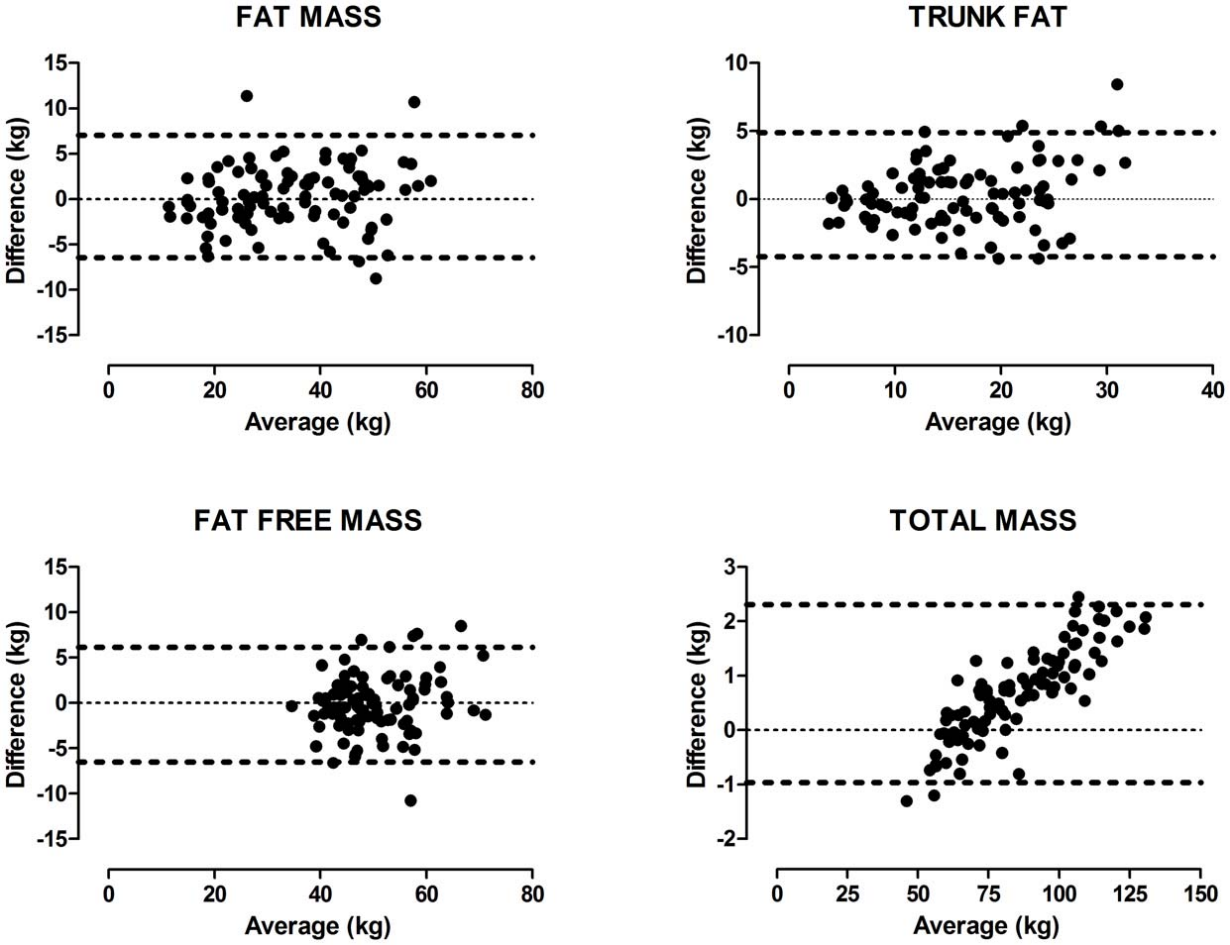
Multiple linear regression models based upon DXA measurements obtained from the Index Group predict the corresponding body masses in the Cross Validation group with good accuracy. A) Bland-Altman analysis of actual versus predicted Total Fat has a very small average bias (i.e., the average of the differences between the two measurements) of 0.281 kg and a standard deviation of 3.44 kg (heavy dashed lines indicate the limits of agreement (mean bias±1.96 its standard deviation): −6.47 to 7.03 kg). B) Predicted Trunk Fat has an average bias of 0.328 (CI: −4.223 to 4.89 kg). C) Predicted Fat Free Mass has a bias of −0.176 kg (CI: −6.49 to 6.14 kg). D) Total Mass (FM + FFM) has an average bias of 0.672 kg (CI: −0.965 to 2.31 kg).

Stepwise multiple regression analysis performed on the Index Group relating the independent anthropometric variables to DXA-derived body composition revealed that significant regressors were age, height, weight, waistline and hipline for FM and FFM, while age, height, weight, and waistline were regressors for TF. These multiple linear regression models predicted the DXA body compartment masses with reasonable precision, as the actual DXA-derived FM and that predicted by the regression model based on anthropomorphic variables were characterized by a mean bias (i.e., actual mass-predicted mass) of 0.281 kg. The accuracy of model estimates for other body compartments were similar (Figure 1B–C), with TF and FFM having mean biases of 0.328 and −0.176 kg respectively. Total body mass (FM + FFM) was extremely well predicted with a mean bias of 0.672 kg and the limits of agreement, i.e., the range over which the difference between the two measurements will fall 95% of the time, was about 3.3 kg (Figure 1D). Notably, unlike the other variables, the bias of the predicted total mass increased with increasing total weight, reaching a maximum of ∼2 kg for weights greater than 100 kg.

**Figure 2:**
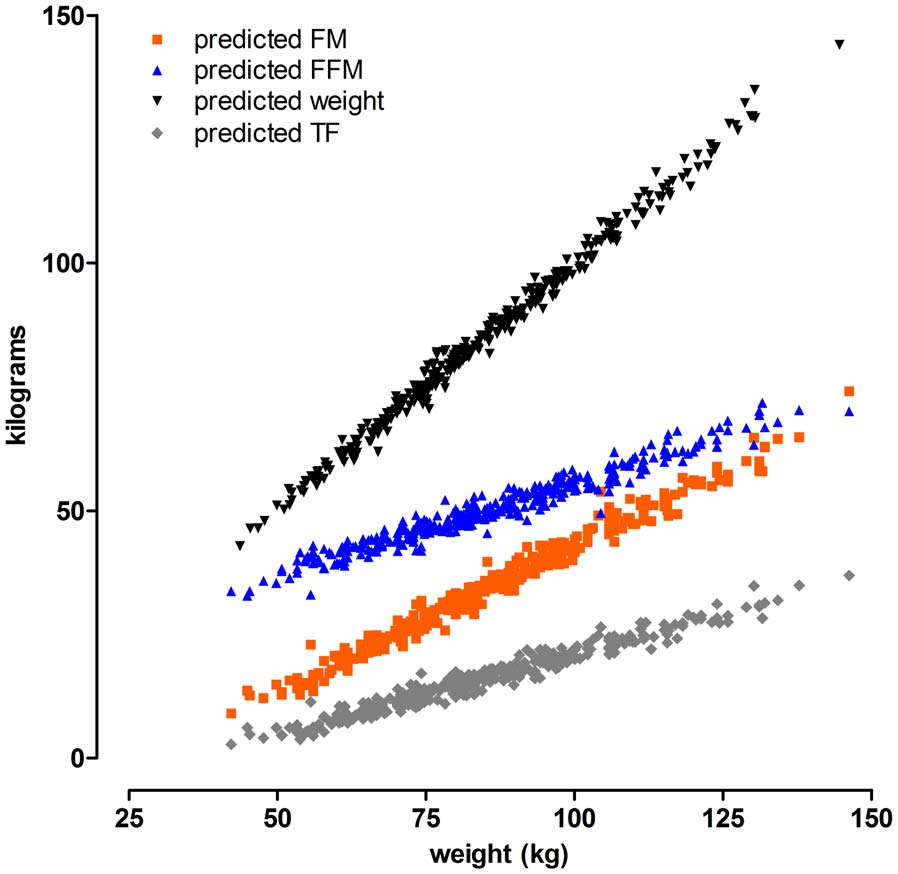
FM increases faster than FFM as a function of weight. Relationships between predicted body composition and weight (abscissa) to DXA-determined values (ordinate) for the full DXA population (IG + CV) are linear and show that with increasing mass, TF and FFM increase at similar rates which are less than for the total FM. Predicted weight is the sum of FFM and FM (see text for discussion).

**Figure 3:**
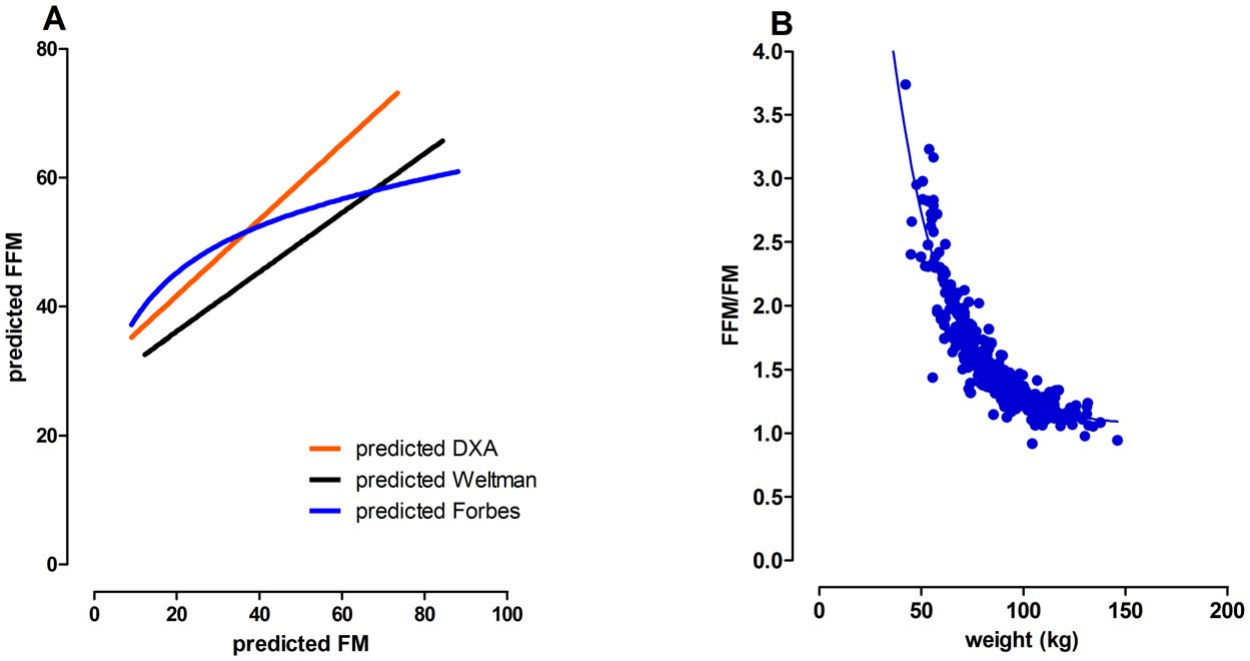
The relationship between the model estimates of FFM and FM is linear. A) Body composition based on modeling DXA-derived data of the full DXA population (IG + CV) using anthropometric variables exhibits a linear relationship between FM and FFM (red line). The anthropometric model of Weltmann [13], derived from hydrostatic weighing in a similar obese female population, is similar (black line). In contrast, data obtained by Forbes [23] for a female population using isotopic dilution methodology is non-linear (blue line). B) The FFM/FM ratio approaches unity for body mass greater than 100 kg.

The relationships between anthropometrically-predicted body composition (regression equations tabulated in Table 4) and weight was explored further. Total weight, as calculated using predicted FM + predicted FFM, matched the actual mass with high accuracy, with the linear regression equation of: DXA weight = 0.63+0.99×predicted weight (r^2^ = 0.99; Figure 2). The contribution of FM and FFM to weight was in a ratio of 0.59 to 0.37, with regression equations FM = 0.59×weight−16.5 (r^2^ = 0.98) and FFM = 0.37×weight+18.7 (r^2^ = 0.961). Trunk fat was related to weight in a 0.32 to 1 ratio (TF = 0.32×weight−11.1; r^2^ = 0.96; Figure 2 D). FFM versus FM was therefore linear with a slope of 0.59 (Figure 3A). The different slopes of the FM and FFM relationships with weight showed that a limiting ratio of approximately 1 was obtained for high body masses (Figure 3B). The relationship between FM and FFM is summarized by the equation: FFM/FM = 12.89(exp^−(0.04×weight)^)+1.06.

**Table 4:** Coefficients of the linear regression equations predicting DXA body composition derived from the Index Group DXA population (n = 241).

	b_0_	b_1_	b_2_	b_3_	b_4_	b_5_
**fat mass (FM)**	−15.49	0.05	0.54	0.11	0	−2.47
**trunk fat (TF)**	−10.14	0.04	0.28	0	0.07	−0.03
**fat free mass (FFM)**	13.96	−0.08	0.41	−0.09	0	7.04

Predicted DXA value = height (**b_0_**)+age (**b_1_**)+weight (**b_3_**)+hipline (**b_4_**)+wa istline (**b_4_**)+**b_5_.**

The equations derived from the linear models of body composition of the IG (predicted FM, TF, and FFM) was subsequently applied to the Cross-Validation population. The means of the predicted values did not differ significantly from actual measurements when compared to the IG (Table 5), validating that the models were also appropriate for this independent test population.

**Table 5:** Body composition of the Cross Validation group is predicted by the regression equations derived from the Index Group.

	FM	TF	FFM
**mean difference** * **(± SD)**	−0.281±3.44 kg	−0.328±2.33 kg	0.176±3.23 kg
**95% CI of mean bias**	−0.98 to 0.42 kg	−0.80 to 0.15 kg	−0.48 to 0.83 kg
**Correlation (r^2^)**	0.965	0.950	0.920
**p value**	NS	NS	NS

*Mean of difference between predicted and actual masses. Statistical significance determined by paired student's t-test.

**Figure 4:**
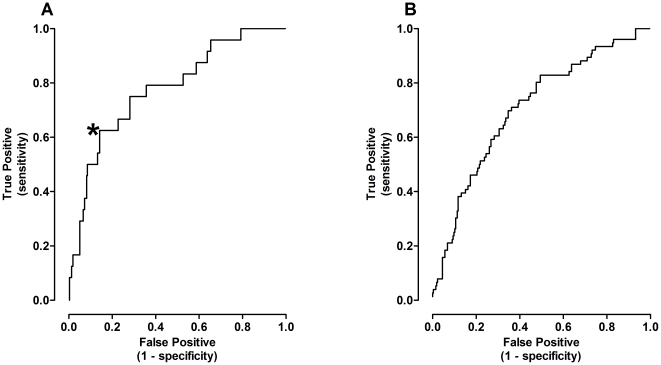
The receiver operating characteristics of a nominal logistical regression using DXA-derived body composition as independent variables to estimate the probability of DM2 or either DM2 or MS in the full DXA population (IG + CV) show that a large percentage of the diseased individuals can be identified without a high cost of a false positive diagnosis. A) The steep slope of the initial portion of the curve to the inflection point (*) indicates that a high proportion of individuals with DM2 can be identifying using DXA-derived FM and TF. Area under the curve is 0.78 with a standard error of 0.05 (24 subjects with DM2 and 327 without; p<0.0001). The 95% confidence interval is 0.68 to 0.88. [Regression equation: risk of DM2 = 1/(1+e^−z^), where z = 1.94−0.53(TF)+0.28(FM).] B) Similarly, the ROC for the detection of either DM2 or MS also shows inflection points, consistent with sensitive detection of subjects with disease. The AUC is 0.71±0.03 (103 subjects with disease, 238 without; p<0.0001). The 95% confidence interval for the AUC is 0.64 to 0.77. [Regression equation: k = 2.32−0.24(TF)+0.09(FM)].

Stepwise nominal logistic regression analysis was then performed using the full data set (IG + CV; n = 341) to determine the probability of an individual diagnosed with DM2 based upon DXA derived body composition. Although FM and TF were highly significant contributing independent variables, FFM was not a significant regressor in this model. The receiver operator characteristic curve (ROC; Figure 4A) constructed using nominal logistic regression exhibited an area under the curve (AUC) of 0.78±0.05 (95% confidence interval 0.68–0.88; p<0.0001). This curve is notable for a steep initial slope of ∼4 (i.e., four times more true positives than false positives identified as the nominal logistic model threshold for a positive diagnosis was reduced) until about 60% of the true positives with a corresponding 15% false positive rate, and was about 0.5 thereafter. Similar to the logistic model estimating the probability of DM2 within the study group, the predictive model of either DM2 or MS within the group depended upon only FM and TF as significant regressors. However, the AUC of the ROC (Figure 4B) was lower at 0.71+0.03 (0.64–0.77; p<0.0001). Like the previous model screening for DM2 alone, the ROC of the model corresponding the presence of either DM2 or MS has a steep initial slope of ∼3.3 to the point 40% true positives and 12% false positives and thereafter reduced to ∼1 until the 80% of true positives are correctly diagnosed.

**Figure 5:**
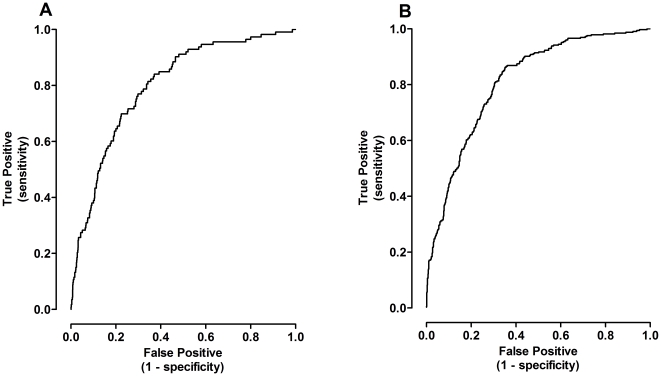
The receiver operating characteristic curve corresponding to a nominal logistical regression using body composition estimated by anthropometric modeling for subjects within the Endocrine Practice group (n = 1153) is consistent with good detection of DM2 or MS. Similar to the DXA population, the ROCs are characterized by initial steep slopes indicating a high detection rate of true positives, but with a low false positive rate. A) The AUC for DM2 detection alone is 0.80±0.02 (1040 subjects negative, 113 positive; p<0.0001). The 95% confidence interval of the AUC is 0.78 to 0.84. [Regression equation: k = 0.14+0.18(Predicted FM)−0.59(Predicted TF)+0.12(Predicted FFM)]. B) The AUC for detecting DM2 or MS is 0.81±0.01 (821 subjects negative, 332 positive; p<0.0001). The 95% confidence interval is 0.78 to 0.84. [Regression equation: k = 0.45+0.33(Predicted FM)−0.83(Predicted TF)+0.06(Predicted FFM)].

A final regression model of body composition was derived using the full data set (IG + CV) with height, age, weight, waistline, and hipline as independent variables. This model was then used to estimate body composition (i.e., FM, TF, and FFM) of a group of females who were a subset of the population of a general endocrinology practice (n = 1135). Nominal logistic regression was performed using predicted FM, TM, and FFM to determine the probability that an individual within the population had metabolic dysfunction. Unlike the smaller DXA group studied, FFM was a significant regressor within the EP population. These analyses produced similar appearing ROCs when compared to the smaller DXA data set. With respect to the probability of the presence of DM2, the ROC (Figure 5A) had an AUC of 0.80±0.02 (0.76–0.84; p<0.0001). Notably, this curve has three inflection points. An initial slope of ∼5.6 up to 28% True Positive (TP) and 5% False Positive (FP). Thereafter, the slope decreased to ∼2.5 until 85% TP and 35% FP. Nominal logistic regression for the probability of DM2 or MS produced an ROC (Figure 5B) with an AUC of 0.81±0.01 (0.78–0.84; p<0.0001). This curve also had a triphasic slope: up to ∼18% TP was associated with a very low rate of FP (0.1%), a slope of ∼18. Thereafter, the slope decreased to ∼2.0 until 90% TP and 35% FP.

## Discussion

In the present work, we have shown that in an obese female population, anthropometric linear regression models predict the DXA-determined body compartments FM, TF, and FFM with good accuracy. Further, the distribution of body fat, as determined by DXA or the anthropometric models, can be used to identify a majority of individuals with a high probability of a formal diagnosis of DM2 or MS with a low false positive rate. To accomplish these, an Index Group was initially studied and the linear models generated were then validated using a similar cross-validation population. A final linear model was derived using data of the full DXA group (IG + CV) and subsequently applied to a larger female population to screen for the presence of DM2 or MS. In this study, we have selected a subject population that is similar in body weight and shape of typical North American females, spanning a wide range of ages (15–80), BMI, and body shapes. The predictive equations obtained are, therefore, valid only for this specific population.

Estimates of body composition based on anthropometric variables have a long history of research interest (reviewed by [14]). Initial investigations were performed using restricted populations for which the models performed well, but not surprisingly, did not generalize to more diverse populations [18]. Subsequently, detailed studies confirmed the logical assumption that anthropometric models are valid if the database employed for analysis is diverse [19], [20]. Earlier studies relied primarily on isotope dilution and specific gravity (hydrostatic) measurements to determine FM and FFM [21] and these did not determine regional differences in fat mass and fat free mass. More recent studies used newer technologies to determine body compartments, e.g., DXA, CT, or MRI, that can also accurately determine regional tissue composition, although with some clear differences among the techniques [22]. Characteristics of regional fat distribution, particularly of centripetal fat, have been identified as important variables in the pathophysiology of metabolic disease and we have modeled anthropometric variables to estimate those body compartments.

Within the populations studied, a number of interesting relationships between weight and body composition were identified. First, FM and FFM are linearly related as a function of weight, such that over a large range of weight, each kilogram is composed of about 60% fat and 40% fat free mass. In contrast, an earlier study using a female population consisting of body fat percentages ranging from undernourished to obese, concluded that the relationship between FM and FFM is a logarithmic one. However, this previous study did not take into account anthropometric variables such as height, age, or body shape [23]. Further, if the outliers who were extremely underweight are excluded from the analysis, logarithmic and linear relationships between FM and FFM become very similar (data not shown). A subsequent study extended the population to include men, but assumed a logarithmic relationship between FM and FFM to develop the model [10]. Neither compare well with the current study, as shown in Figure 3A. In contrast, an anthropometric study of an obese female population similar to ours [13] concluded that a linear relationship exists between FM and FFM, although with a shallower slope (0.46±0.01 versus 0.59±0.01 in the present study respectively). The reason for this discrepancy is unclear, but could depend upon the technology used to determine body composition. For example, different methodologies to determine body composition in obese women are not equivalent when each is compared to the “gold standard” four compartment model [22].

Results of the present study also show that the relationship between FM and FFM is such that as body-weight increases, the contributions of FM and FFM eventually become approximately equal. This has physiological relevance because resting metabolic rate is proportional to FFM. Individuals of greater weight will have a corresponding greater fat free mass and thus will be more likely to lose weight while undergoing calorie restriction. Although height and body shape correlated well with FFM, partial correlation analysis shows that only the waistline is appreciably correlated to FFM when the other variables are held constant. Age was related to FFM inversely and to TF in a positive manner. The lack of correlation of age with other anthropometric variables suggests that there are other variables not accounted for by the model, e.g., possibly age-related differences in metabolic rate. Regional fat distribution did weakly correlate with FM as increases in hipline were associated with greater FM. Not surprisingly, TF was correlated with waistline and negatively with height.

A number of longitudinal studies have evaluated the use of simple anthropometrics, e.g., waistline, waist-hip ratio, and others, in populations to predict the risk of developing DM2 in the future [24]. In our approach, we were interested in identifying individuals within an overweight population that are likely to have undiagnosed metabolic dysfunction at the time of screening. Based on studies that have confirmed the importance of central fat distribution for the development of metabolic dysfunction, including DM2, we have used DXA-derived body composition in logistic regression models to determine the probability of the presence of disease. Beginning with the DXA data group, the discrimination of the logistic function derived from DXA data is quite good with an AUC of 0.78 (Figure 4A). This is superior to the results of screening based on questionnaires and compares reasonably well with population screening based on clinical data obtained invasively. For example, the KORA survey carried out in Austria used a variety of published DM2 screening questionnaires which were administered to 1573 participants aged 55–74 and DM2 established by oral glucose tolerance testing. The resulting AUCs range from 0.61–0.67 [25]. In contrast, in the San Antonio Heart study screening was based on a predictive model that included fasting glucose levels to assess for diabetes had an AUC of 0.84 [26]. Identification of DM2 or MS by use of other variables that were collected invasively, e.g., hemoglobin A1c, do not appear to improve the sensitivity of detecting disease. For example, data from the Health Aging and Body Composition Study provides AUCs <0.7 when used for diagnosis of DM2 [27].

The inflection points of the ROCs in the current study are of interest, as the steep slopes in the left portion of the curves show that within the population a large fraction of individuals with DM2 can be identified using anthropometric linear models to estimate body composition, without incurring an unacceptably high false positive rate. For example, for the threshold corresponding to the point of the curve identified by an asterisk in Figure 4A, 62.5% of the individuals with DM2 are correctly characterized (15 of 24), whereas only 14% of people without DM2 are incorrectly identified as positive (45 of 317) and 86% of individuals without disease are correctly identified, i.e., a sensitivity of 62.5 and a specificity of 86 percent. Not surprisingly, the shape of the ROC for logistic regression identifying individuals with either DM2 or MS was less steep, as would be expected in an obese population in which individuals with disease overlapped with others who are more similar than different in their anthropometric characteristics.

Use of regression models to study the larger EP population confirmed the relevance of this analytical approach. However, a primary limitation of this study is that it is valid only in the population evaluated. This group is not representative of a general population, consisting as it does of mostly middle aged, Caucasian, overweight females that have consulted an endocrinologist. On the other hand, such a population would also be expected to be more difficult to differentiate individuals with disease from normals, as the subjects are more similar in their body characteristics than individuals within a more heterogeneous population. The results of this proof-of-concept investigation suggests that larger studies performed using more generalized population databases could lead to the development of simple screening tools to identify those at high risk for metabolic dysfunction. The incorporation of other non-invasive, easily and inexpensively obtained variables, e.g., previously-validated questionnaires, would likely improve the sensitivity and specificity of the models. A final caveat is that this model cannot predict future risk of DM or MS, but only the likelihood that an individual presently has significant risk for having disease. It is this utility that especially suggests that this approach might be very useful in mass health screening scenarios.

## Methods

In accordance with the Unites States Code of Federal Regulations 7 C.F.R. § 1c.101b4, this research is exempt from review by an Institutional Review Board as it constitutes “Research, involving the collection or study of existing data, documents, records, pathological specimens, or diagnostic specimens, if these sources are publicly available or if the information is recorded by the investigator in such a manner that subjects cannot be identified, directly or through identifiers linked to the subjects.” The patient populations studied were drawn from an ambulatory, general endocrinology and weight management practice. Informed consent was not obtained as all data were analyzed retrospectively in an anonymous manner. The initial study population consisted all females in the medical practice for whom complete anthropometric data were available who underwent DXA for a weight control-fitness program (n = 341). A second independent group consisted of all female patients in a single general endocrine practice (EP) for whom full anthropometric data were available. The patients were clinically stable and except for diabetes and metabolic syndrome, did not have known active diseases that potentially could affect body composition (e.g., uncorrected thyroid dysfunction). All data were collected by a single investigator (RY) and were blinded as to personal identity for data analysis. The DXA scanner utilized was a Delphi QDR series (Hologic, Bedford, MA). Data included in this analysis consisted of total body mass (TBM), total fat mass (FM), trunk (abdominal) fat mass (TF), and fat free mass (FFM). Anthropometric variables were obtained to the same level of precision as likely would be obtained within a mass health screening environment. Specifically, subjects were weighed to within 0.25 kg using a precision clinical balance while lightly clothed and without footwear. Height was measured to within 0.25 cm using a clinical stadiometer, waistline (circumference to 0.5 cm, taken at the level of the umbilicus at the end of gentle expiration), hipline (circumference at the level of the greater trochanter), and age. Diagnosis of DM2 was based upon the criteria of the American Diabetes Association [28] and MS was established following the criteria of the National Cholesterol Program [3].

The group of 341 individuals who underwent DXA scanning was randomly divided into a Cross-Validation group (CV; n = 95) and the remaining 246 subjects constituted an Index Group (IG). Characteristics of the subject populations are summarized in Table 1. A predictive model for DXA-derived body composition of the IG was then generated using stepwise multiple regression analysis using FM, TM, and FFM as dependent variables and anthropometric measurements as independent variables. The resulting multiple regression models were then applied to the CV group to determine how well the IG regression equations predicted DXA body composition of these individuals. Using the DXA-derived body composition of the fulI (G + CV) group, a stepwise nominal logistic regression model was also constructed to predict the probability that an individual within the DXA data set had metabolic disease. Following successful validation of the models in the two groups, a final multiple regression model was determined using the entire data set (n = 341) to estimate body composition and regional fat distribution of a larger female population drawn from a general Endocrinology Practice (EP; n = 1153). Finally, these values were then used to generate a stepwise nominal logistic regression model to predict the presence of metabolic disease.

All statistical analyses were performed using JMP (SAS Inc, Cary, NC). Dependent variables were DXA-determined FM, TF, and FFM. Independent variables were age, height, weight, waistline, and hipline. The relationships between the independent and dependent variables were examined for evidence of non-linearity by calculating Pearson correlation coefficients for each independent variable. Each variable was also transformed by log10(x), 1/x, and squareroot (x). The observation that there were no significant differences observed for the correlation coefficients between the untransformed and transformed variables and the dependent variables assured linearity. Of note, the results of prior research examining anthropometric variables and body density-derived fat and lean body masses have shown that there is no significant curvilinearity between variables, confirming the appropriateness of the use of linear modeling [29]. Partial correlation coefficients were calculated to estimate the correlation contributed by each pair of variables when all other variables were held constant. A comparison of actual versus predicted values was accomplished using the Bland-Altman method [30] that determines bias (actual-predicted) as a function of the average of the actual and predicted values estimated by two different methods. Nominal logistic regression was performed to assess the sensitivity and specificity of the diagnosis of DM2 or MS in the groups. Probability values were calculated from a two-tailed distribution and p<0.05 was considered significant.

## References

[1] Eckel RH, Grundy SM, Zimmet PZ (2005). The metabolic syndrome.. Lancet.

[2] Ford ES, Giles WH, Dietz WH (2002). Prevalence of the metabolic syndrome among US adults: findings from the third National Health and Nutrition Examination Survey.. JAMA.

[3] Mottillo S, Filion KB, Genest J, Joseph L, Pilote L (2010). The metabolic syndrome and cardiovascular risk a systematic review and meta-analysis.. J Am Coll Cardiol.

[4] Lindstrom J, Peltonen M, Eriksson JG, Aunola S, Hamalainen H (2008). Determinants for the effectiveness of lifestyle intervention in the Finnish Diabetes Prevention Study.. Diabetes Care.

[5] Stolar MW (2010). Defining and achieving treatment success in patients with type 2 diabetes mellitus.. Mayo Clin Proc.

[6] Fonseca VA (2008). Identification and treatment of prediabetes to prevent progression to type 2 diabetes.. Clin Cornerstone.

[7] Ilanne-Parikka P, Eriksson JG, Lindstrom J, Peltonen M, Aunola S (2008). Effect of lifestyle intervention on the occurrence of metabolic syndrome and its components in the Finnish Diabetes Prevention Study.. Diabetes Care.

[8] Diabetes Prevention Program (2006). Relationship of body size and shape to the development of diabetes in the diabetes prevention program.. Obesity (Silver Spring).

[9] Bray GA, Jablonski KA, Fujimoto WY, Barrett-Connor E, Haffner S (2008). Relation of central adiposity and body mass index to the development of diabetes in the Diabetes Prevention Program.. Am J Clin Nutr.

[10] Mingrone G, Marino S, DeGaetano A, Capristo E, Heymsfield SB (2001). Different limit to the body's ability of increasing fat-free mass.. Metabolism.

[11] Forbes G (2003). Some adventures in body composition, with special reference to nutrition.. Acta Diabetol.

[12] Matsuo T, Douchi T, Nakae M, Uto H, Oki T (2003). Relationship of upper body fat distribution to higher regional lean mass and bone mineral density.. J Bone Miner Metab.

[13] Weltman A, Levine S, Seip RL, Tran ZV (1988). Accurate assessment of body composition in obese females.. Am J Clin Nutr.

[14] Jackson AS (1984). Research design and analysis of data procedures for predicting body density.. Med Sci Sports Exerc.

[15] Albanese CV, Diessel E, Genant HK (2003). Clinical applications of body composition measurements using DXA.. J Clin Densitom.

[16] Andreoli A, Scalzo G, Masala S, Tarantino U, Guglielmi G (2009). Body composition assessment by dual-energy X-ray absorptiometry (DXA).. Radiol Med.

[17] Leslie WD, Ludwig SM, Morin S (2010). Abdominal fat from spine dual-energy x-ray absorptiometry and risk for subsequent diabetes.. J Clin Endocrinol Metab.

[18] Foster C, Jackson AS, Pollock ML, Taylor MM, Hare J (1984). Generalized equations for predicting functional capacity from treadmill performance.. Am Heart J.

[19] Jackson AS, Pollock ML, Ward A (1980). Generalized equations for predicting body density of women.. Med Sci Sports Exerc.

[20] Tran ZV, Weltman A (1989). Generalized equation for predicting body density of women from girth measurements.. Med Sci Sports Exerc.

[21] Forbes GB (2002). Perspectives on body composition.. Curr Opin Clin Nutr Metab Care.

[22] Minderico CS, Silva AM, Keller K, Branco TL, Martins SS (2008). Usefulness of different techniques for measuring body composition changes during weight loss in overweight and obese women.. Br J Nutr.

[23] Forbes GB (2000). Body fat content influences the body composition response to nutrition and exercise.. Ann N Y Acad Sci.

[24] Han TS, Feskens EJ, Lean ME, Seidell JC (1998). Associations of body composition with type 2 diabetes mellitus.. Diabet Med.

[25] Rathmann W, Martin S, Haastert B, Icks A, Holle R (2005). Performance of screening questionnaires and risk scores for undiagnosed diabetes: the KORA Survey 2000.. Arch Intern Med.

[26] Stern MP, Williams K, Haffner SM (2002). Identification of persons at high risk for type 2 diabetes mellitus: do we need the oral glucose tolerance test?. Ann Intern Med.

[27] Lipska KJ, De Rekeneire N, Van Ness PH, Johnson KC, Kanaya A (2010). Identifying dysglycemic states in older adults: implications of the emerging use of hemoglobin A1c.. J Clin Endocrinol Metab.

[28] American Diabetes Association (2010). Standards of medical care in diabetes–2010.. Diabetes Care.

[29] Weltman A, Seip RL, Tran ZV (1987). Practical assessment of body composition in adult obese males.. Hum Biol.

[30] Bland JM, Altman DG (1986). Statistical methods for assessing agreement between two methods of clinical measurement.. Lancet.

